# *TTN*-related hereditary myopathy with early respiratory failure presented with elevated hemoglobin initially: A case report and literature review

**DOI:** 10.1016/j.heliyon.2024.e29637

**Published:** 2024-04-12

**Authors:** Hanyang Liang, Dong Liu, Qian Gao, Zhenguo Zhai

**Affiliations:** a Department of Pulmonary and Critical Care Medicine, Center of Respiratory Medicine, China-Japan Friendship Hospital, National Center for Respiratory Medicine Institute of Respiratory Medicine, Chinese Academy of Medical Sciences National Clinical Research Center for Respiratory Diseases, Beijing 100029, China; b State Key Laboratory of Cardiovascular Disease, Fuwai Hospital, National Center for Cardiovascular Diseases, Chinese Academy of Medical Sciences and Peking Union Medical College, Beijing 100037, China; c Peking University China-Japan Friendship School of Clinical Medicine, Beijing 100029, China

**Keywords:** Respiratory failure, Hereditary myopathy, Diaphragmatic dysfunction, TTN, Case report

## Abstract

**Background:**

As common abnormal conditions in clinical practice, hypoxemia and respiratory failure are mainly caused by various respiratory diseases. However, other causes are easily overlooked but deserve more attention from doctors.

**Case presentation:**

A 44-year-old man presented with dyspnea for 10 years. In the early stage, his dyspnea was mild without hypoxemia, and he was misdiagnosed with polycythemia vera due to elevated hemoglobin level. He later developed to respiratory failure but he did not have weakness in his extremities. The positional difference in pulmonary function tests and arterial blood gas analysis led us to identify the respiratory muscle dysfunction. Fatty infiltration of the thigh muscle found by magnetic resonance imaging and muscle biopsies gave us more clues to the causes of diaphragmatic dysfunction. Finally, in combination with his family history and the results of whole exome sequencing, he was diagnosed with hereditary myopathy with early respiratory failure (HMERF, OMIM 603689) caused by a variant in the titin gene (*TTN*).

**Conclusions:**

We have identified a Chinese family with HMERF due to genetic variants in *TTN* NM_001256850.1: c.90272C > T, p. Pro30091Leu, located at g.179410829A > G on chromosome 2 (GRCh37)*,* which may be specifically associated with the diagrammatic dysfunction. And hyperhemoglobinemia could serve as a potential sign for the early identification of HMERF.

## 1 Introduction

Hypoxemia is not rare in clinical practice. As the disease progresses, hypoxemia could develop to respiratory failure, whose common causes include alveolar hypoventilation, diffusion abnormalities, increased oxygen consumption, and ventilation-perfusion mismatch. Besides, rare causes such as respiratory muscle weakness could lead to alveolar hypoventilation. Due to the specialization of pulmonary and critical care medicine (PCCM), pulmonologists usually screen for common respiratory diseases, but other causes are easily ignored, including the inability of central nervous system, peripheral nerve, neuromuscular junction and muscle [1]. These uncommon causes may be noticed because of an exacerbation or even remain unrecognizable. After excluding the usual causes, myopathies should be included in the differential diagnosis, and the possible reasons include metabolic disorders, inflammation, toxicity, and inheritance [2]. Here, we report a Chinese patient who presented with exertional dyspnea without muscle symptoms in the extremities, and he was finally diagnosed with hereditary myopathy with early respiratory failure (HMERF).

HMERF was first described as a separate nomological entity by Lars Edström and coworkers in 1990 [3]. In the following years, only scattered sporadic cases and single families were reported, mainly belonging to Northern European populations [4]. With the increasing popularity of whole exome sequencing (WES) in clinical practice, more and more cases have been reported worldwide, including the United Kingdom, Italy, France, Portugal, Germany, Argentina and Japan [5]. In 2015, the first HMERF from China was reported by Huashan Hospital [6], and so far 11 Chinese patients have been reported by several neuromuscular centers [7]. Our case is the first reported patient diagnosed in the Department of PCCM. Through this retrospective study, we aimed to improve the awareness of pulmonologists and provide some signs for the early recognition of HMERF.

## 2 Case presentation

**Fig. 1:**
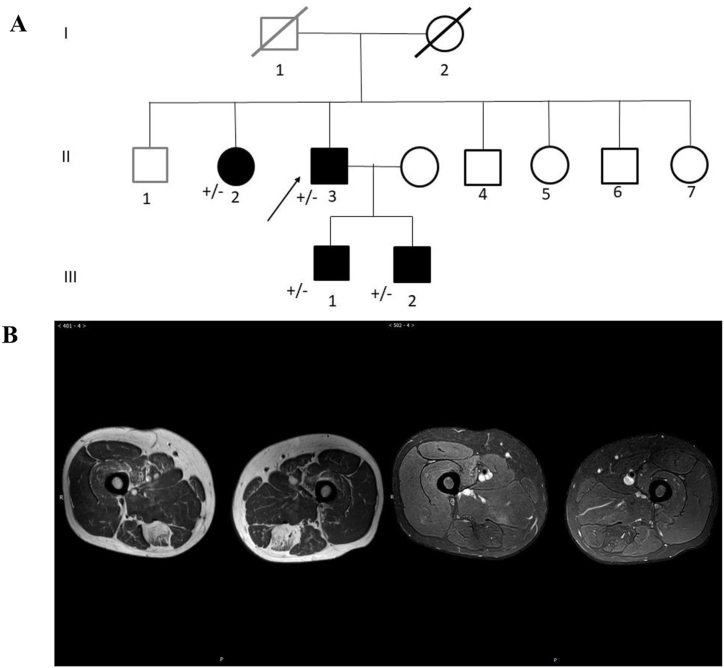
(A) Pedigree of the family: the patient (II-3), his brother (II-1) who had cardiac enlargement and his sister (II-2) who had short of breath after activity; (B) Fatty infiltration on thigh muscle in magnetic resonance imaging of TW1 and STIR.

The proband is a 44-year-old man presenting with a 10-year history of unprovoked shortness of breath and chest tightness during heavy exercise or in the supine position, but without limitation of daily activities initially. At the first admission in 2012, his hemoglobin (Hb) was found to be elevated to 199 g/L (normal range: 120–160g/L) with normal partial pressure of oxygen (PO_2_) in arterial blood gas analysis (ABG), and no cytological abnormalities were observed in the specimen of bone marrow with a negative JAK2V167F gene mutation. He was diagnosed with polycythemia vera and was treated with interferon (the dose and duration were unknown), but the symptoms did not improve with treatment. At the second admission in 2013, his Hb was elevated to 201 g/L and the ABG revealed a PO_2_ of 63 mmHg and partial pressure of carbon dioxide (PCO_2_) of 52 mmHg. No abnormalities were observed on echocardiography, computed tomography pulmonary angiogram (CTPA) or electromyography (EMG). α–glucosidase, β–galactosidase, cardiolipin antibody, anti-neutrophil cytoplasmic antibody, hepatic and renal function were all normal. He was diagnosed with secondary polycythemia due to hypoxemia. His symptoms improved after the use of a non-invasive ventilator. Afterwards, he was admitted to our department with exacerbated exertional dyspnea in 2021. His physical examination revealed no obvious abnormalities. He had been a smoker for 30 years with a 13-year history of hypertension. His elder sister had similar symptoms as him, being short of breath after physical activity, and his father died of cardiac enlargement. The pedigree of the proband is displayed in Fig. 1A.

**Fig. 2:**
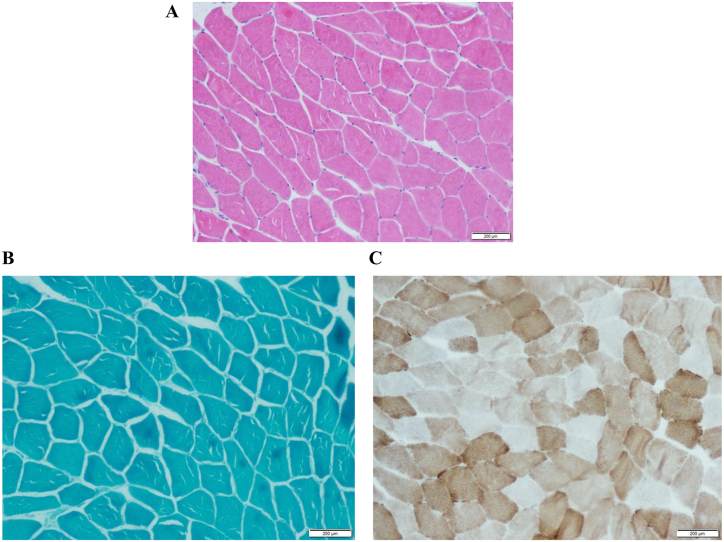
The results of patients' histopathological biopsy of biceps brachii. No abnormal performances were found in histological staining. (A) H&E staining: Within the fascicle, there is no evidence of atrophy, degeneration, necrosis, or regenerating muscle cells. There is no apparent infiltration of inflammatory cells around the endomysium, perimysium, and blood vessels. (B) Modified Gomori Trichrome (MGT) staining: No fragmented red fibers and muscle fibers with rimmed vacuoles. (C) Cytochrome *c* Oxidase Staining (COX) staining: No COX-negative muscle fibers.

Some abnormal results of the admission examinations were as follows: creatine kinase-MB (CK-MB) 5.63 ng/mL (normal range: <3.61 ng/mL), Hb 187 g/L (normal range: 115–150g/L), total bilirubin (TBIL) 50.96 μmol/L (normal range: <23.00 μmol/L), direct bilirubin (DBIL) 11.95 μmol/L (normal range: <8.00 μmol/L). His immunoglobulin G level was slightly elevated, but his immunoglobulin G4 level was normal, and the serum anti-smooth muscle antibody was weakly positive. Pulmonary function tests revealed forced vital capacity (FVC) 1.43 L (35.4 %), forced expiratory volume in first second (FEV1) 1.12 L (33.6 %), FEV1/FVC 0.78, total lung capacity (TLC) 2.66 L (43.7 %), residual volume (RV) 1.14 L (59.9 %), RV/TLC 0.42. ABG on room air in supine position revealed low PO_2_ (49 mmHg) and high PCO_2_ (69 mmHg) compared to standing position (PO_2_:54 mmHg, PCO_2_:62 mmHg), while FVC in the supine position (0.77 L) is significantly lower than standing position (1.43 L). The maximum inspiratory pressure was 4.90 kPa (normal range: 11.8 ± 3.63 kPa for adult males), which was 45.5 % of the predicted value, and the maximum expiratory pressure was 8.08 kPa (normal range: 13.2 ± 2.94 kPa for adult males), which was 58.2 % of the predicted value. Diaphragmatic ultrasound showed right diaphragm mobility of 1.7 cm and left diaphragm mobility of 1.9 cm in maximum inspiratory phase, the right diaphragm mobility of 0.5 cm, and left diaphragm mobility of 0.3 cm in normal breathing (normal range: > 2cm), indicating bilateral reduced diaphragmatic activity. In addition, reverse movement on both sides and thickened parasternal intercostal muscles were observed. Abdominal ultrasound showed fatty liver. No valvular regurgitation and pulmonary hypertension were found on echocardiography. No significant abnormalities were observed in brain magnetic resonance imaging (MRI), cervical spine MRI, EMG of peripheral muscle. Fatty infiltration of the thigh muscle was found on muscle MRI in T1-weighted imaging and short time of inversion recovery (Fig. 1B). No abnormalities were found in the biopsy of biceps brachii (Fig. 2).

**Fig. 3:**
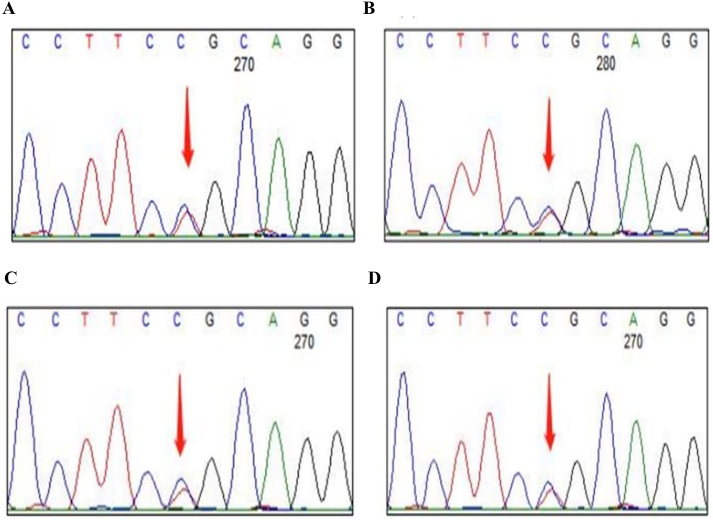
GES results of the patient (A), his sister (B) and his two asymptomatic sons (C and D) which carried heterozygous variation of *TTN* c.90272C > T, p. Pro30091Leu locus.

WES was performed on the proband using the Agilent SureSelect Human All Exon V6 Kit (Agilent Technologies, Santa Clara, CA, USA) and HiSeq X Ten Reagent kit V2.5 (Illumina, U.S.A). The brief protocol is provided in the supplementary material 1. A heterozygous variant of *TTN* NM_001256850.1: c.90272C > T, NP_001243779.1: p. Pro30091Leu (also referred to as NM_001267550.2: c.95195C > T, NP_001254479.2: p. Pro31732Leu and NM_003319.4: c.68000C > T, NP_003310.4: p. Pro22667Leu [[7], [8], [9]]) was identified in the proband. The genomic location of it is g.179410829A > G on chromosome 2 (GRCh37). Sanger sequencing of this variant also identified the proband's elder sister with similar symptoms (II2 in Fig. 1A) and two asymptomatic sons (III1 and III 2 in Fig. 1A) as heterozygous carriers. WES indicated that he and his two sons as well as his sister carried heterozygous variation of *TTN* c.90272C > T, p. Pro30091Leu (Fig. 3A, 3B, 3C, 3D).

Complete blood count and ABG were done for his sons. His 5-year-old son had hyperhemoglobinemia (Hb 153 g/L) [normal range: 120–140 g/L] with normal PO_2_, and his 14-year-old son had normal hemoglobin and hypoxemia (PO_2_ 76.7 mmHg). Based on clinical manifestations, investigations, family history and WES, he was diagnosed with HMERF. Non-invasive ventilator was continued and he received respiratory rehabilitation and acupuncture. Our patient experienced less discomfort than before, but no obvious difference in ABG was observed at discharge. In December 2023, the patient was followed up by telephone, his symptoms had not worsened and the ventilator parameters had not been adjusted.

## 3 Discussion

Our patient was a middle-aged man with the onset of mild dyspnea and elevated hemoglobin and his symptoms had progressively exacerbated with hypercapnia. For differential diagnosis, obstructive hypoventilation was excluded based on the results of unreduced FEV1/FVC and no signs of emphysema on chest computerized tomography (CT). Restrictive hypoventilation was identified with the results of decreased TLC and RV. Therefore, pulmonary and airway diseases were excluded according to the results of chest CT and pulmonary function tests. It was necessary to change the diagnostic direction from common respiratory diseases. To our surprise, the results of pulmonary function varied in different positions, which was consistent with the results of ABG. And we started to pay more attention to diaphragmatic dysfunction, which was demonstrated by ultrasound [10].

For diaphragmatic dysfunction, hyperhemoglobinemia could be the only abnormal laboratory result due to chronic hypoxic compensations at early stage. Evidences of central and peripheral nervous system diseases were not found by head and neck MRI. In the differential diagnosis of myopathies, our patient had hyperCKemia but hypercapnia due to respiratory muscle weakness is not a common complication of inflammatory myopathy. Besides, he had no rash, myalgia, limb weakness and no significant abnormalities in the antinuclear antibody profile. Hereditary motor sensor neuropathy is characterized by weakness in the distal lower limbs, leg deformities, and loss of tendon reflexes without myogenic injury. And the diagnosis of peripheral neuropathy could not be supported by EMG findings. As for metabolic myopathy, muscle biopsy did not show morphological alterations of abnormal mitochondria, glycogen or lipid metabolism with normal α–glucosidase, β–galactosidase. Considering his family history and symptoms of respiratory insufficiency, hereditary myofibrillar myopathy was taken into account, and classical pathological changes were not observed under light microscope, suggesting us to apply GES for seek other potential causes.

With the help of GES, a heterozygous variant was detected within the *TTN* gene in the subject, and the c.90272C > T, p. Pro30091Leu variant was located within the exon 293 of the *TTN* gene. The *TTN* c.90272C > T, p. Pro30091Leu variant causes the amino acid 30,091 of the encoded protein to be mutated from proline to leucine. Bioinformatics software such as SIFT, Polyphen, MutationTaster, and others predicted this mutation to be deleterious for the gene or its product (PP3). Analysis based on the EXAC database and gnomAD databases revealed the highest population frequency for this variant as 0.0000178279 (PM2_Supporting). Through the verification of the subject and relatives, both the patient and his sister carried the heterozygous variant locus, so this variant has the possibility of familial co-segregation [11]. Our patient, along with his family members, carried the heterozygous substitution *TTN* p. Pro30091Leu, which is a verified pedigree of HMERF with this variant in China. In addition, *TTN* p. Pro30091Leu has been previously reported heterozygous in HMERF patients [[5], [6], [7], [8], [9],^12^,^13^], including a *de novo* occurrence [6], and homozygous in at least 2 more severely affected HMERF patients [5,14] (Table 1). This variant also seems to segregate with HMERF in at least 3 affected relatives with relatively mild symptoms from 3 families [5,8], although heterozygous asymptomatic carriers are also observed [5,9,12], probably indicating a reduced penetrance. Moreover, this variant is present at a relatively low frequency (rs753334568, 3/282,182 chromosomes, 1 from South Asian and 2 from non-Finish European) in the general population according to the Genome Aggregation Database (gnomAD). Multiple computational prediction tools including SIFT, MutationTaster and REVEL (0.727) predict a damaging effect to the protein. *In vitro* functional studies indicate this variant may be destabilizing and impair the solubility of titin probably by disturbing the proper protein folding [14,15]. Taken together, this *TTN* variant can be classified as a likely pathogenic according to American College of Medical Genetics and Genomics (ACMG) guidelines [16](PM2_Supporting, PP3, PS2_Moderate, PS4_Moderate, PP1, PS3_Moderate).

**Table 1:** Symptoms and laboratory findings of patients with p. Pro30091Leu mutation.

Patient（Nationality）	Age/sex	Symptoms		Laboratory findings			Reference
		First symptom (age)	Muscle involvement	CK (IU/L)	Respiratory function	EMG	
A（Britain）	67/M	Difficulty in lifting lower limbs (30s)	Proximal UL, Proximal ll, neck flex, calf hypertrophy	204	FVC 52 % sitting	N. A	[12]
B（Italian）^a^	40/M	Nocturnal hypoventilation, exertional dyspnea (30)	Neck flexors, Proximal LL Ankle dorsiflexion	Normal	CO2-retention, FVC 39 %	Myopathic	[5]
C（French）^a^,^b^	44/M	Effort Breathlessness (27)	Wheelchair bound at age 36. Proximal UL, LL, Fingers extensors, Distal LL	274	Assisted ventilation with tracheostomy	Myopathic	[5]
D（French）^b^	61/M	Respiratory failure (42)	No limb weakness	Normal	Non-invasive night-time ventilation FVC 60 %	Myopathic	[5]
E（French）^b^	66/F	Hypoventilation (53)	Neck flexors, No limb weakness	Normal	Non-invasive night-time ventilation VC 45 %	N. A	[5]
F（Chinese）	29/M	Difficulty in bending the neck and spine (adolescence)	Scapular winging, spinal rigidity, neck flexor and knee flexor, calf hypertrophy,	630	FVC 19 % of predicted value and PaCO2 65 mmHg	myopathic	[6]

CK, creatine kinase; EMG, electromyography; FVC, forced vital capacity; LL, lower limbs; N. A, not assessed; UL, upper limbs.

a Patient B and C are homozygous, others are heterozygous.

b Patient C, D and E has Portuguese ancestry. Patient C is sporadic, while D and E are brothers of one family.

WES identified a heterozygous *TTN* p. Pro30091Leu variant located in the A band of titin previously associated with HMERF [[6], [7], [8],[11], [12], [13],^15^,^17^] in the proband, as well as in his elder sister with similar symptoms and two asymptomatic sons (Fig. 1A), indicating a possible co-segregation for this variant. No more suspicious rare variants related to myopathy were identified. Similarly, the family screening from previous reports also identified several heterozygous asymptomatic carriers, including the heterozygous parents of homozygous probands [5,9,12,14]. Compared with the reported heterozygous patients with no symptoms [5,9,12,14] or relatively late-onset (>40 years) mild symptoms (including our proband and his sister, II2 and II3 in Fig. 1A), homozygous ones tended to be more severely affected, with earlier onset (<31 years) and more rapid progression [5,14], which may be due to the deleterious effect on *TTN* p. Pro30091Leu on the protein folding and solubility [14,15]. These observations indicate the variable expressivity and reduced penetrance of autosomal dominant HMERF, or maybe a more appropriate saying for semi-dominant disease for this variant. Moreover, the existence of an undetectable intronic *TTN* variant contributing to the relatively more severe symptoms observed in the proband could not be excluded due to the detection limitations of WES itself.

HMERF is an autosomal dominant inheritance disease with a variant of the *TTN* gene encoding titin, a sarcomeric protein that spans from the Z-disc to the M-band [18]. Titin plays crucial functional and structural roles in the sarcomere, while a variant of titin could cause titinopathy, a group of inherited muscle diseases with high clinical and genetic heterogeneity. In HMERF, all patients harbor a variant located in exon 343 in *TTN* that codes for the fibronectin III domain 119 (FN3 119) in the 10th motif of the 11-element motif A-band super-repeat [19]. Until now, fifteen disease-causing variants have been recorded in the Human Gene Mutation Data [7]. Previous biological research revealed that the p. Pro30091Leu variant in A150/Fn3 119 was recessive, which became penetrant when a cis titin kinase variant worked in synergy with protein turnover pathways [20]. From the clinical perspective, patients with the p. Pro30091Leu variant often manifest as normal or mild respiratory insufficiency. Consequently, it is still under controversy whether the p. Pro30091Leu variant is a recessive or a semidominant variant.

The clinical manifestations of titinopathy vary and people in all ages could be affected. It manifests as slowly progressive distal or proximal muscle weakness, diaphragmatic weakness, early respiratory failure, and may involve the myocardium, respiratory muscles, peripheral nerves, joints and gastrointestinal tract. Currently, no specific treatment is available for HMERF. Non-invasive ventilation is suitable for most patients, and Bilevel Positive Pressure Airway with average volume-assured pressure support mode may be preferred [21]. With the implementation of next-generation sequencing into clinical practice, the diagnostic rate of titinopathy is rapidly improving these years [22].

Muscle imaging is fairly vital for the diagnosis of HMERF. Typical manifestations of MRI include fatty infiltration of the semitendinosus muscle and anterior compartment muscles of the lower legs. Other involved sites include the gluteus minimus at the pelvic level, the sartorius at the thigh muscle and the tibialis posterior at the lower leg level [7]. Notably, the use of MRI is also reported to help discover new families or sporadic cases and testing oligosymptomatic individuals [23]. In our study, the findings of muscle MRI were in accordance with reported typical manifestations, and ultrasonography was used to help detect both the thickness and functions of respiratory muscles as well. In muscle specimens of HMERF, the remarkable pathological feature is multiple cytoplasmic bodies (CBs) and rimmed vacuolar in fibers at subsarcolemmal [5]. The above features may not be found in every patient because of milder presentation (Table 2). In Uruha's study, the sensitivity of necklace cytoplasmic bodies was 82 %, which means the possibility of false-negative possibility existed [24]. In our case, we didn't find any abnormalities in fiber size, central nuclei or fiber splitting in perimysial and endomysial connective tissue, which may be due to the variable expression of the mutated allele in different cells in one patient. Whether the biopsies were properly targeted also limited the sensitivity of muscle biopsies [13]. Because of the patchy nature of abnormalities in myofibrillar myopathies, abnormal muscle pathology is not present in all affected individuals.

**Table 2:** Muscle imaging findings and histology of patients with p.Pro30091Leu mutation.

Patient (Nationality）	Muscle Imaging^a^	Muscle Histology	Reference
A（Britain）	N. A	Deltoid: Dystrophic features, rimmed vacuoles, fiber splitting, few fibers showing dark blue areas on Gomori trichrome	[12]
B（Italian）	Glma, Glme, Glmi, Ilps, Obt, RF, VL, VI, VM, Sa, Gr, SM, ST, AM, AL, BF, TA, EH, ED, Pr	Fiber-size variation, increase of internal nuclei, necrosis, rimmed vacuoles, cytoplasmic bodies	[5]
C（French）	Glma, Glme, Glmi, Ilps, Obt, RF, VL, VI, VM, Sa, Gr, SM, ST, AM, AL, BF, TA, EH, ED, Pr, Gm, Gl, S, TP, FP	Fiber-size variation, increase of internal nuclei, necrosis, rimmed vacuoles, cytoplasmic bodies	[5]
D（French）	N. A	Fiber-size variation, increase of internal nuclei, rimmed vacuoles, cytoplasmic bodies	[5]
E（French）	SM, BF	Fiber-size variation, increase of internal nuclei, rimmed vacuoles, cytoplasmic bodies	[5]
F（Chinese）	SM, Pr	Nonspecific myopathic changes	[6]

AL, adductor longus; AM, adductor magnus; BF, biceps femoris; ED, extensor digitorum longus; EH, extensor; hallucis longus; FP, flexor hallucis and digitorum longus; Gl, gastrocnemius lateralis; Glma, gluteus maximus; Glme, gluteus medius; Glmi, gluteus minimus; Gm, gastrocnemius medialis; Gr, gracilis; Ilps, iliopsoas; LL, lower limbs; N. A, not assessed; Obt, obturator; Pr, peroneus longus; RF, rectus femoris; S, soleus; Sa, sartorius; SM, semimembranosus; ST, semitendinosus; TA, tibialis; anterior; TP, tibialis posterior; UL, upper limbs; VI, vastus intermedius; VL, vastus lateralis; VM, vastus medialis.

a Muscle imaging findings refers to fatty degenerative changes in magnetic resonance imaging.

There is a lack of specific indicators for the early identification for the HMERF. After reviewing the natural history of our patient, the initial elevated hemoglobin did not attract the doctor's enough attention due to the normal PO2. The same phenomenon observed in his son further supported the hypothesis that elevated hemoglobin could serve as an alarming sign for HMERF in patients with unexplained dyspnea. In addition, the finding of mildly elevated hemoglobin in the patient's early routine examination before the onset of respiratory symptoms confirmed our hypothesis.

However, there were some limitations in our study. Although we proposed a hypothesis and validated it at the phenotypic level, we did not perform a deeper validation in terms of the transcriptome and metabolome. The patient's sons were still asymptomatic, and further follow-up is necessary.

## 4 Conclusions

Due to the specialization of PCCM, pulmonologists tend to make differential diagnosis within their own disciplines, delaying early diagnosis and treatment. Hypoxemia and respiratory failure are common in practice but associated with a variety of potential causes. After excluding pulmonary and airway diseases, other abnormalities affecting the respiratory muscles deserve more attention. Extra-pulmonary signs could help clinicians tell the causes, including unexplained elevated hemoglobin and hypoxemia in asymptomatic family members. We have identified a Chinese family with HMERF due to genetic variants in *TTN* NM_001256850.1: c.90272C > T, p. Pro30091Leu, located at g.179410829A > G on chromosome 2 (GRCh37), which likely affected the function of diaphragm.

## Availability of data and materials

The research data are not deposited into publicly accessible repositories. The datasets used and analyzed in this study are available from the corresponding authors upon reasonable request.

## Ethics approval and consent for publication

Our study has received approval from the ethics committee of China-Japan Friendship Hospital.

## Consent for publication

Written informed consent for publication of clinical details and clinical images were obtained from the patient.

## Funding sources

This study is fund by Chinese Academy of Medical Sciences Innovation Fund for Medical Sciences (CIFMS) (2020-I2M-B-095, 2021-I2M-1-001, 2021-I2M-1–049).

## Disclosure of conflict of interest

The authors declare that the research was conducted in the absence of any commercial or financial relationships that could be construed as a potential conflict of interest.

## CRediT authorship contribution statement

**Hanyang Liang:** Writing – review & editing, Writing – original draft, Methodology, Investigation, Formal analysis, Data curation, Conceptualization. **Dong Liu:** Writing – review & editing, Writing – original draft, Software, Resources, Project administration, Methodology, Investigation, Formal analysis, Data curation. **Qian Gao:** Visualization, Validation, Funding acquisition. **Zhenguo Zhai:** Visualization, Validation, Supervision, Funding acquisition.

## Declaration of competing interest

The authors declare that they have no known competing financial interests or personal relationships that could have appeared to influence the work reported in this paper.
