# Supplementary material for: TTN-related hereditary myopathy with early respiratory failure presented with elevated hemoglobin initially: A case report and literature review
Source: Heliyon. 2024 Apr 12;10(8):e29637. doi: 10.1016/j.heliyon.2024.e29637 (PMC11035038; doi:10.1016/j.heliyon.2024.e29637)
Supplement: Multimedia component 1 [file mmc1.docx]

Supplementary material 1. Brief Protocol of Whole Exome Sequencing in Our Research

We collected venous blood from probands and their relatives, all of whom signed informed consent forms. Genomic DNA was extracted from the samples, and whole exome sequencing (WES) was performed on the probands. After the analysis results were obtained, suspicious pathogenic sites were validated through Sanger sequencing, and screening was conducted in family samples. The simplified steps were as follows: We used the Covaris ultrasonic disruptor to randomly fragment 300ng of DNA into fragments of approximately 150-200bp. Then, we used the SureselectXT Enrichment System kit (Agilent, USA) for library construction and capture. Finally, we performed paired-end sequencing on the HiSeq X10 sequencer (Illumina, USA) using the Illumina cBOT cluster generation system and HiSeq PE Cluster kit. We applied bioinformatics techniques to annotate sequencing data, including alignment to population databases (dbSNP and ExAC) and disease databases (ClinVar, OMIM, HGMD, Cosmic, etc.), and software predictions of site pathogenicity (SIFT, Polyphen-2, MutationTaster, CADD, etc.), with variant site filtering based on a minimum allele frequency (MAF) of ≤0.05. According to the interpretation guidelines for general variant sites released by the American College of Medical Genetics and Genomics (ACMG) in 2015 and subsequent supplementary updates for specific evidence (PVS1, PM3, PM2), as well as specific interpretation rules for MYH7, Rasopathy, and other myocardial disease-related factors, we comprehensively assessed the pathogenicity of the sites. We performed Sanger sequencing validation and specific screening for selected (potentially) pathogenic variant sites in probands and their direct relatives. All experimental procedures strictly adhered to the manufacturer's instructions and standard protocols, in accordance with the ethical requirements of the China-Japan Friendship Hospital Ethics Committee.
